# MicroRNA‐146b‐5p promotes atrial fibrosis in atrial fibrillation by repressing TIMP4

**DOI:** 10.1111/jcmm.16985

**Published:** 2021-10-13

**Authors:** Qing Ye, Quan Liu, Xiaolong Ma, Shuyun Bai, Pengfei Chen, Yichen Zhao, Chen Bai, Yang Liu, Kemin Liu, Meng Xin, Caiwu Zeng, Cheng Zhao, Yan Yao, Yue Ma, Jiangang Wang

**Affiliations:** ^1^ Department of Cardiac Surgery Beijing Anzhen Hospital Capital Medical University Beijing China; ^2^ Department of Cardiothoracic Surgery Benq Hospital Affiliated Hospital of Nanjing Medical University Nanjing China; ^3^ Key Laboratory of Interdisciplinary Research Institute of Biophysics Chinese Academy of Sciences Beijing China; ^4^ Department of Cardiac Surgery Fuwai Hospital National Center for Cardiovascular Diseases Chinese Academy of Medical Sciences and Peking Union Medical College Beijing China; ^5^ Center for Cardiac Intensive Care Beijing Anzhen Hospital Capital Medical University Beijing China; ^6^ Department of Cardiology Beijing Anzhen Hospital Capital Medical University Beijing China; ^7^ Medical School of University of Chinese Academy of Sciences Beijing China; ^8^ Guangzhou Regenerative Medicine and Health Guangdong Laboratory Guangzhou China

**Keywords:** atrial fibrillation, fibrosis, matrix metalloproteinases, metalloproteinases inhibitor 4, microRNA

## Abstract

Alteration of tissue inhibitors of matrix metalloproteinases (TIMP)/matrix metalloproteinases (MMP) associated with collagen upregulation has an important role in sustained atrial fibrillation (AF). The expression of miR‐146b‐5p, whose the targeted gene is TIMPs, is upregulated in atrial cardiomyocytes during AF. This study was to determine whether miR‐146b‐5p could regulate the gene expression of TIMP4 and the contribution of miRNA to atrial fibrosis in AF. Collagen synthesis was observed after miR‐146b‐5p transfection in human induced pluripotent stem cell‐derived atrial cardiomyocytes (hiPSC‐aCMs)‐fibroblast co‐culture cellular model *in vitro*. Furthermore, a myocardial infarction (MI) mouse model was used to confirm the protective effect of miR‐146b‐5p downregulation on atrial fibrosis. The expression level of miR‐146b‐5p was upregulated, while the expression level of TIMP4 was downregulated in the fibrotic atrium of canine with AF. miR‐146b‐5p transfection in hiPSC‐aCMs‐fibroblast co‐culture cellular model increased collagen synthesis by regulating TIMP4/MMP9 mediated extracellular matrix proteins synthesis. The inhibition of miR‐146b‐5p expression reduced the phenotypes of cardiac fibrosis in the MI mouse model. Fibrotic marker MMP9, TGFB1 and COL1A1 were significantly downregulated, while TIMP4 was significantly upregulated (at both mRNA and protein levels) by miR‐146b‐5p inhibition in cardiomyocytes of MI heart. We concluded that collagen fibres were accumulated in extracellular space on miR‐146b‐5p overexpressed co‐culture cellular model. Moreover, the cardiac fibrosis induced by MI was attenuated in antagomiR‐146 treated mice by increasing the expression of TIMP4, which indicated that the inhibition of miR‐146b‐5p might become an effective therapeutic approach for preventing atrial fibrosis.

## 1 INTRODUCTION

Atrial fibrillation (AF) is the most common arrhythmia with increasing prevalence and indidence over the last decade.^1^, ^2^ Electrical and structural remodelling have been implicated in the pathogenesis of AF, and atrial fibrosis is the hallmark of structural remodelling.^3^ The fibrotic process is a complex process that involves increased production and activity of a variety of extracellular matrix (ECM) proteins secreted by fibroblasts, including enzymes that modify the ECM such as matrix metalloproteinases (MMPs) and tissue inhibitors of MMPs (TIMPs). Numerous studies have suggested that an alteration of TIMP/MMP associated with the collagen upregulation plays an important role in progressing sustained AF.^4^, ^5^, ^6^, ^7^

MicroRNAs (miRNAs) are endogenous ~23‐nt RNAs that can complementarily bind to messenger RNAs (mRNAs) of protein‐coding genes and lead to their regulation post‐transcriptionally.^8^, ^9^ The human miR‐146 family has two genes: miRNA‐146a and miRNA‐146b. These two miRNAs, which located on different chromosomes, have different regulatory functions.^10^ Most published studies have focussed on their effect on the immune system or in cancer, while only a few reported their role in cardiovascular disease.^11^, ^12^, ^13^, ^14^ In our previous study, 10 differentially expressed miRNAs and 624 differentially expressed mRNAs are identified and 1 miRNA‐target gene pair, that is miR‐146b‐5p and TIMP4 from atrial cardiomyocytes (CMs), was constructed in the left atrial appendage (LAA) from patients with nonvalvular paroxysmal AF (NVPAF). Furthermore, we found that the 3‐UTR region of TIMP‐4 region has only 1 highly conserved miR‐146b‐5p binding site, and the ability of miR‐146b‐5p to repress the expression of TIMP‐4 was further verified by Western blotting analysis in mouse cardiac fibroblasts transfected with miR‐146b5p. However, the role of miR‐146b‐5p on atrial fibrosis remains mysterious.^15^This study hypothesized that miR‐146b‐5p could target the gene and contribute to the balance between TIMP4 and MMP9, which is associated with atrial fibrosis. We verified whether miR‐146b‐5p upregulation participates in the induction of collagen expression in human induced pluripotent stem cell‐derived atrial cardiomyocytes (hiPSC‐aCMs) fibroblast contact co‐culture system. Furthermore, a myocardial infarction (MI) mouse model was used to verify the effect of miR‐146b‐5p downregulation on cardiac fibrosis.

## 2 MATERIALS AND METHODS

The expansion of the method section can be found in the Supplemental Materials.

### 2.1 HiPSC derived atria‐like cardiomyocytes

An early conducted research has documented a highly homogenous atrial‐like cardiomyocytes (aCMs) obtained from human pluripotent stem cells.^16^, ^17^ Details are shown in the Supplemental Materials.

### 2.2 hiPSC‐aCMs‐fibroblast contact co‐culture and rapid electrical field stimulation

After 24 h, the hiPSC‐aCMs and fibroblasts were in the confluent phase and grew in monolayers, while the hiPSC‐aCMs were still in spontaneous synchronized contraction. After 6 h of serum‐free culture, the cells were moved into an incubator containing an atmosphere of air enriched with 5% CO2 at 37°C (Figure S1) and a rapid electrical field stimulation (RES) was conducted. Details are available in the Supplemental Materials.

### 2.3 Animals

All animal studies were done in compliance with the regulations and guidelines of Capital Medical University institutional animal care and conducted according to the AAALAC and the IACUC guidelines. All the C57BL/6 mice (6–8 weeks old, weighing 20–25 g) were housed in an environment with a temperature of 24 ± 1ºC, a relative humidity of 50 ± 1% and a light/dark cycle of 12/12 h and fed with food and water *at libitum*. Twelve healthy male Beagles, weighing between 8 and 12 kg, were individually housed in stainless steel cages from the time of arrival until euthanasia. Beagle dogs were separately housed in cages at a temperature of 13–16˚C, a relative humidity of 40–70% and a light/dark cycle of 12/12 h and fed with water and high‐quality adult dog food (3–5% of its weight) twice a day.

### 2.4 Canine model of AF

Male Beagles were randomly divided into two groups: sham control (*n* = 6) and atrial tachypacing (ATP, *n* = 6) group (Figure S2). Details are available in the Supplemental Materials.

### 2.5 Myocardial infarction (MI) wild‐type mice

MI was induced in 10‐week‐old wild‐type (WT) C57BL/6 mice by permanent ligation of the left anterior descending coronary artery (LAD). Details are available in the Supplemental Materials.

### 2.6 Induction of AF in wild‐type mice

Intracardiac pacing was performed in wild‐type C57BL/6 mice by inserting an eight‐electrode catheter (1.1F, octapolar EP catheter, Scisense) through the jugular vein and advancing it into the right atrium and ventricle. The inducibility of atrial arrhythmias was tested by applying 6‐s bursts through the catheter electrodes using an automated stimulator that was part of the data acquisition system. The first 6‐s burst had a cycle length of 40 ms, decreasing in each successive burst with a 2‐ms decrement down to a cycle length of 20 ms. Then, 50 ng/kg carbachol was injected through the inner canthus veniplex. Two minutes later, the same combination of bursts was applied to the animals. Successful induction of AF was defined as a period of rapid, irregular atrial rhythm lasting at least 1 s.

### 2.7 Statistical analysis

All results were stated as mean ±standard deviation of the mean for continuous variables, and categorical variables were stated as the number of patients and percentage. Differences between the groups were analysed using the Student *t* test or one‐way analysis of variance for continuous variables and the chi‐square test for categorical variables. All tests were two‐tailed, and a *P*‐value of 0.05 was statistically significant. The data were analysed in GraphPad Prism 8.0 (GraphPad Software Inc.).

## 3 RESULTS

### 3.1 Increased miR‐146 expression in a canine AF model induced by atrial tachypacing

**FIGURE 1:**
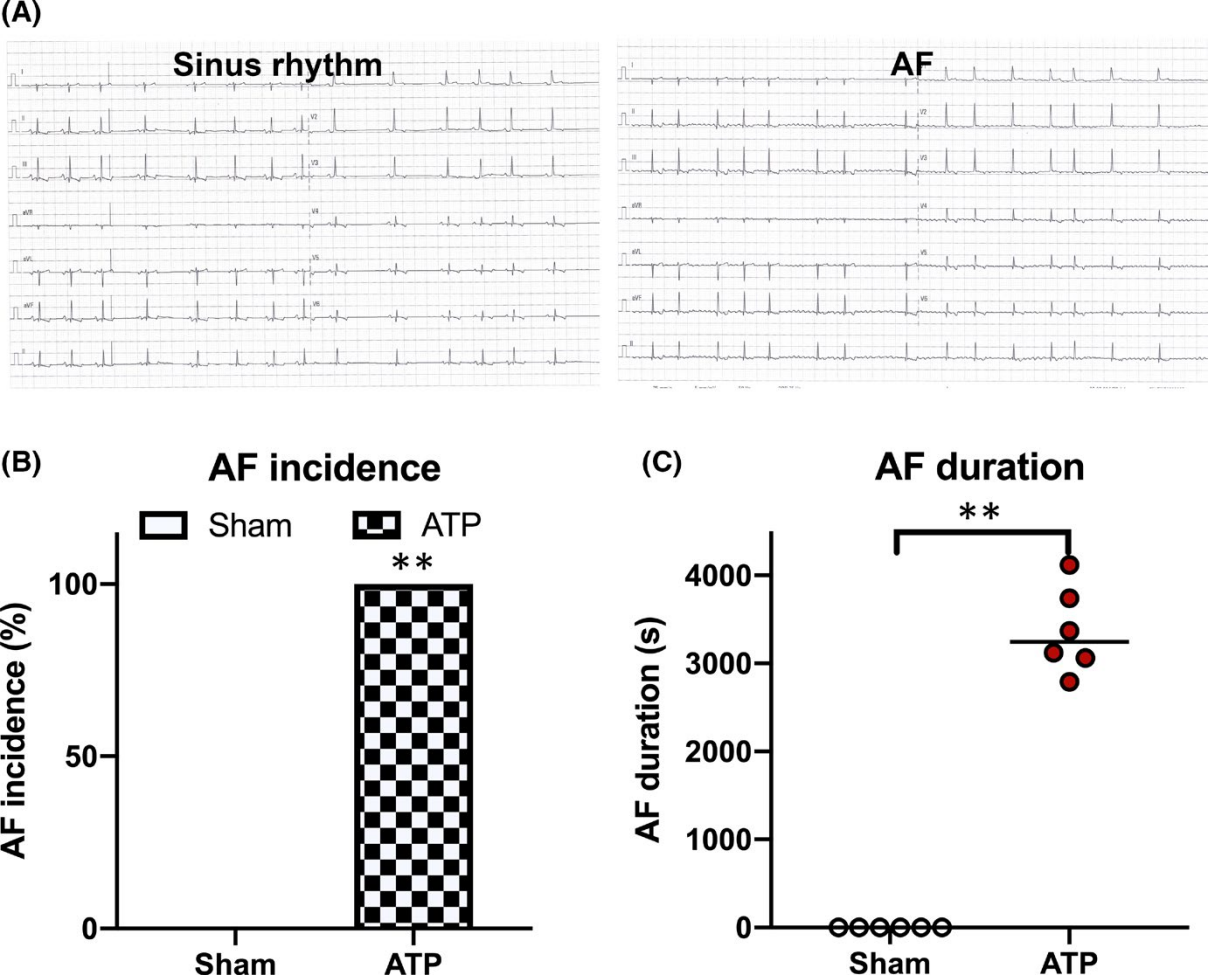
A, Representative surface ECG showing AF in an ATP dog and sinus rhythm in a sham dog. Surface ECG showing the lack of P waves and irregular RR intervals. B and C, AF inducibility and duration expanded substantially in ATP dogs. ^**^ *p* < 0.001 versus sham. AF, Atrial fibrillation

Based on the previous results, we decided to examine ATP in AF canine recapitulated atrial fibrosis phenotypes and the increased expression of miR‐146b‐5p. No spontaneous AF was observed in the sham group. However, a typically induced atrial tachycardia by programmed electrical atrial pacing was inducible with average 3345 ± 146 s AF duration in all ATP canines (*p *< 0.0001 versus sham, Figure 1A–C).

**FIGURE 2:**
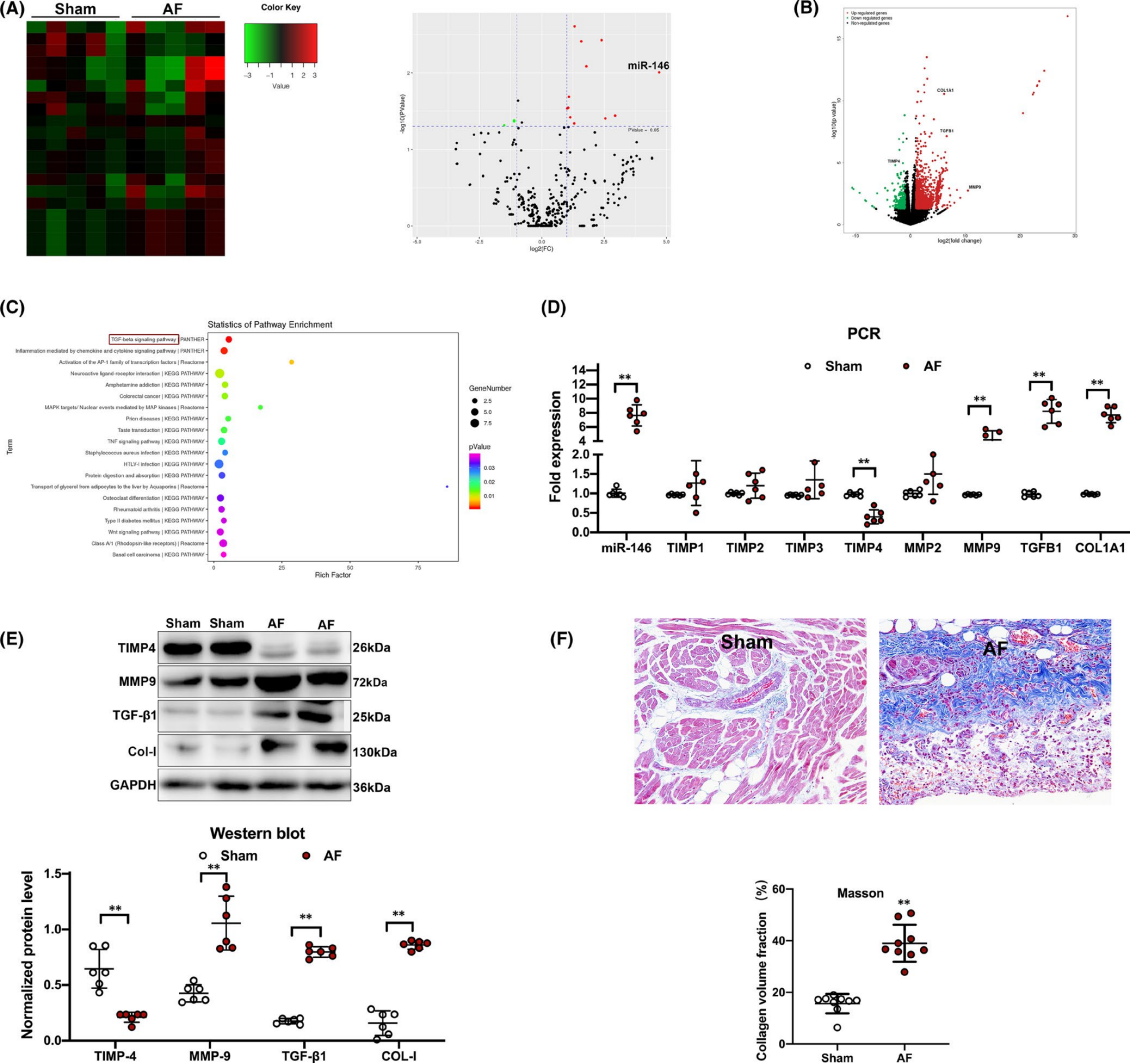
A, Hot map and volcano plot showed that miR‐146b‐5p is the most pronounced change among the 20 differentially expressed miRNAs in atrial cardiomyocytes from dogs with AF. B, The volcano plot showed fibrotic genes were significantly dysregulated in atrial cardiomyocytes from dogs with AF. C, Pathway enrichment analysis predicted the TGF‐β1pathway was the most significant involved pathway based on the mRNA profiling results. D, PCR analysis showed a significant upregulation of miR‐146b‐5p, MMP9, TGFB1and COL1A1, with a significant downregulation of TIMP4 expression in dogs with AF. E, Western blot analysis showed protein expression of TIMP4, MMP9, TGF‐β1 and COL‐I in atrial cardiomyocytes from dogs with AF and sinus rhythm. F, Histopathological changes and the collagen volume fraction in left atrium after staining with Masson's trichrome stain from dogs with AF and sinus rhythm. ^**^ *p* < 0.001 versus sham. AF, Atrial fibrillation

Next, miRNA and mRNA expression profiles of the left atrium from canines in the AF (*n* = 5) and sham groups (*n* = 5) were compared. The miRNA expression profiles revealed that 20 miRNAs, including previously described miRNA (such as miR‐146b‐5p, miR‐19, miR‐142 and miR‐155), were differentially expressed between two groups (>twofold change; *p* < 0.05) (Table 1). A mRNA microarray was used to assay the gene expression profiles. We observed a upregulation of expression of 1174 genes and a downregulation of the expression of 218 genes in canines with AF (*p* < 0.05; Table [Link], [Link]). In particular, miR‐146b‐5p and fibrosis‐related genes (TIMP4, MMP9, TGFB1 and COL1A1) indicated the significance in change among the expressed miRNAs and mRNAs respectively. In Figure 2A,B, the volcano plot has illustrated an elevation that is more than four times of the expression in canines with AF compared with shams. The expression was elevated more than 4 times in canines with AF, comparing with shams that is clearly stated in the volcano plot shown in Figure 2A,B. Pathway enrichment analysis predicted that the transforming growth factor‐β1 (TGF‐β1) pathway was the most predominant involved pathway based on the mRNA profiling results (Figure 2C). Real‐time quantitative reverse‐transcription PCR (RT‐PCR) analysis confirmed our microarray results and showed a significant upregulation of miR‐146b‐5p, MMP9, TGFB1and COL1A1, with a significant downregulation of TIMP4 expression (Figure 2D). The associated protein expression of TIMP‐4, MMP9, TGF‐β1 and COL‐I was further verified with Western blot analysis (Figure 2E). Furthermore, we examined histopathological changes and the collagen volume fraction in the left atrium after staining with Masson's trichrome stain. There was a grand increase in collagen volume fraction in the AF canines compared with the sham group (Figure 2F).

**TABLE 1:** MiRNAs differentially expressed in cardiomyocytes between canines with atrial fibrillation and sinus rhythm

miRNAs	Log_2_^(AF/Sham)^	*p*‐value
Upregulated^a^		
cfa‐miR‐146b‐5p	4.708167	0.009828
cfa‐miR‐19	2.943996	0.036217
cfa‐miR‐142	1.787493	0.008199
cfa‐miR‐155	2.549247	0.039337
cfa‐miR‐127	2.549245	0.039328
cfa‐miR‐134	1.587698	0.003875
cfa‐miR‐136	1.301447	0.045827
cfa‐miR‐150	1.318164	0.002465
cfa‐miR‐217	1.064136	0.028541
cfa‐miR‐324	1.143566	0.038083
cfa‐miR‐369	1.015712	0.029181
cfa‐miR‐376a	1.091215	0.020535
cfa‐miR‐379	3.087363	0.001201
cfa‐miR‐381	2.398528	0.003733
cfa‐miR‐758	3.087369	0.001188
Downregulated^a^		
cfa‐miR‐411	−1.10622	0.041943
cfa‐miR‐432	−1.10618	0.042216
cfa‐miR‐485	−1.10623	0.041852
cfa‐miR‐487b	−1.10605	0.043109
cfa‐miR‐493	−1.5056	0.048414

^a^ Expression levels of miRNAs are described as upregulated or downregulated in AF relative to those in the sham group.

### 3.2 Rapid depolarization of hiPSC‐aCMs‐fibroblast co‐culture cellular model

**FIGURE 3:**
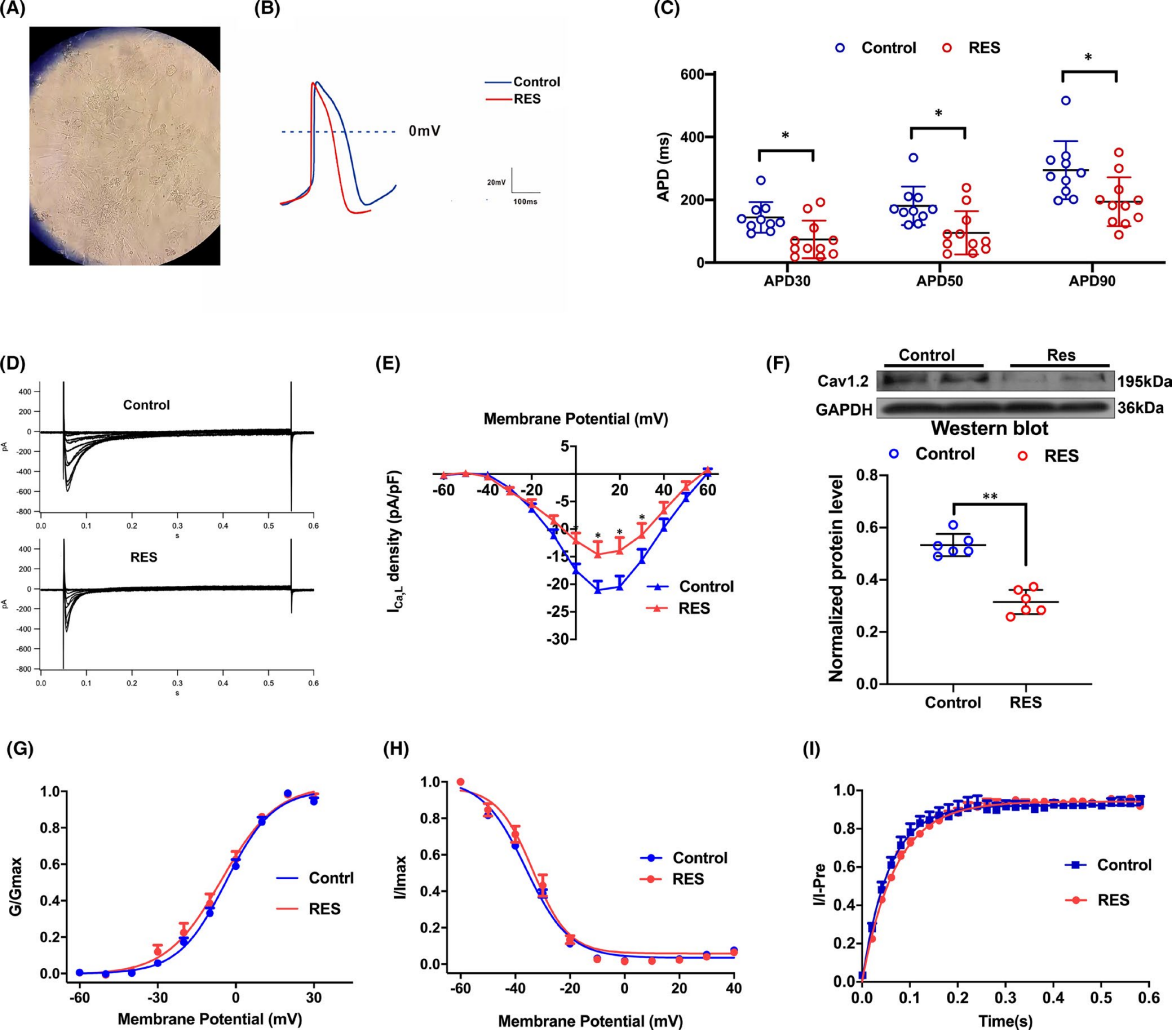
A, hiPSC‐aCMs‐fibroblast contact co‐culture. B and C, Decreased action potential period was found in hiPSC‐aCMs after RES. D‐F, *I_Ca_*_,_ *_L_* traces, present density‐voltage linkage, and Cav1.2 expression critically reduced after RES. G‐I, Regularized e *I_Ca_*_,_ *_L_* activation, inactivation and recovery curve. RES, rapid electrical field stimulation

The characterization of hiPSC‐aCMs was described before.^17^ Figure 3A shows the hiPSC‐aCMs‐fibroblast contact co‐culture. The beating of myocytes was crucial when distinguishing the hiPSC‐aCMs from fibroblasts (Supplementary Video). Next, we tested whether co‐culture could change electrophysiological properties in hiPSC‐aCMs and fibrosis‐related mRNA expression in the fibroblasts. We found that the action potential of hiPSC‐aCMs and action potential duration (APD) had no changes after 24 h co‐culture (Figure S3A) The identical mRNA expression of miR‐146b‐5p, TIMP4, MMP9, TGFB1 and COL1A1 (Figure S3B) was confirmed by the PCR analysis.

When using RES, we found that the APD of hiPSC‐aCMs was markedly shortened, which was consistent with the atrial remodelling process during AF (Figure 3B,C). Looking into the effects of RES on electrophysiologic properties of hiPSC‐aCMs, the current densities of inward L‐type Ca^2+^ current (*I_Ca_*_,_ *_L_*), transient outward K^+^ current (*I_to_*), ultrarapid delayed rectifier K^+^ current (*I_Kur_*) and sodium current (*I_Na_)*, that leads to the varied phases of the action potential in hiPSC‐aCMs, were recorded. As illustrated for individual cells, a 24‐h period of RES caused a marked reduction in *I_Ca_*_,_ *_L_* (with no modification of the activation/inactivation and recovery time course) (Figure 3D‐I). These findings demonstrate that downregulation of *I_Ca_*_,_ *_L_*, a hallmark of AF remodelling *in vivo*, also occurs in hiPSC‐aCMs paced in culture. Notably, after RES, there was no change in the current densities of *I_to_*, *I_Kur_* and *I_Na_* in hiPSC‐aCMs (Figure S4).

### 3.3 MiR‐146b‐5p transfection in hiPSC‐aCMs induced increased interstitial collagen by decreasing the expression of TIMP4 in the co‐cultured cellular model

Seventy‐two hours after miR‐146b‐5p transfection, a study of miR‐146b‐5p by qRT‐PCR for mRNA levels is conducted on hiPSC‐aCMs, which reflected that the miR‐146b‐5p inhibitor could critically decrease the expressionism of miR‐146b‐5p in CMs (Figure S5).

**FIGURE 4:**
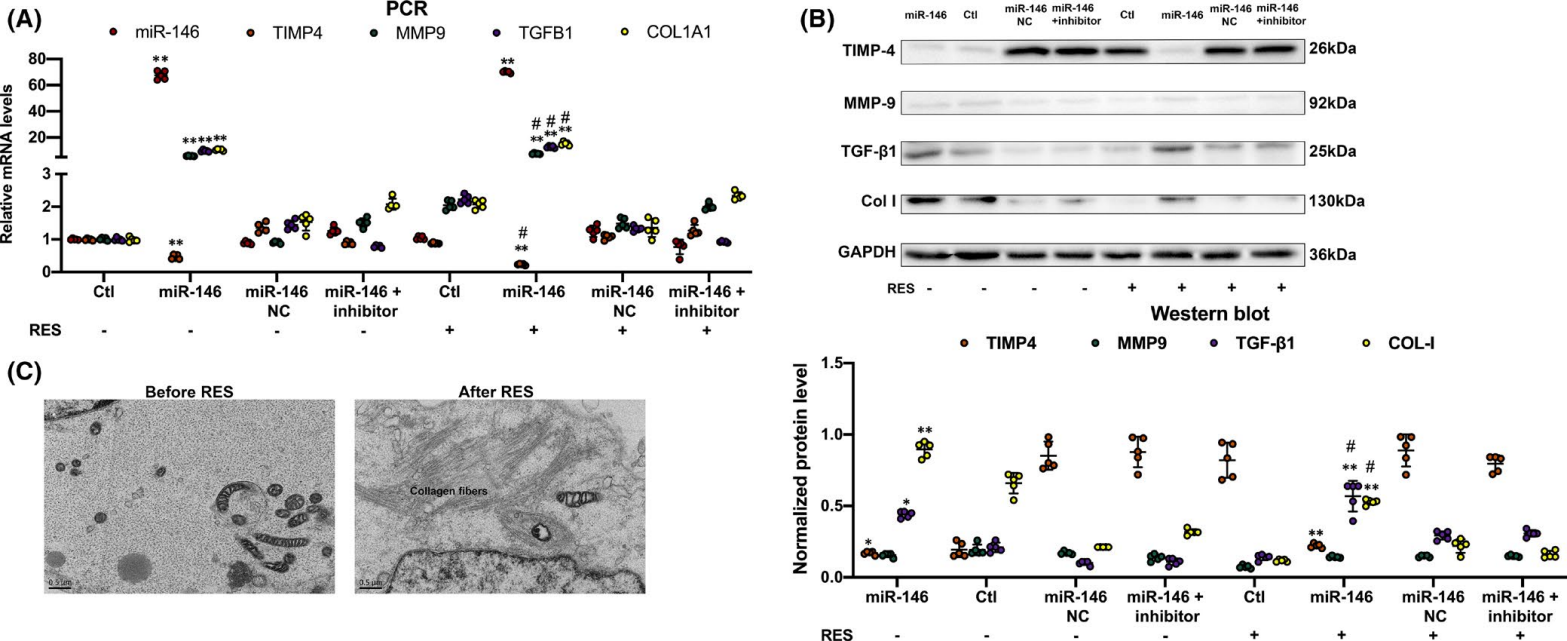
A, Quantitative RT‐PCR analysis showed mRNA expression changes in hiPSC‐aCMs‐fibroblast contact co‐culture model after transfection of miR‐146b‐5p with or without RES. B, Western blot analysis showed protein expression in hiPSC‐aCMs‐fibroblast contact co‐culture model after transfection of miR‐146b‐5p with or without RES. C, The collagen fibres were accumulated in extracellular space after rapid depolarization of miR‐146b‐5p overexpressed hiPSC‐aCMs‐fibroblast co‐culture cellular model by RES (X4000). ^*^ *p* < 0.05 versus control; ^**^ *p* < 0.001 versus control; ^#^ *p* < 0.001 versus RES (‐). NC, negative control; RES, rapid electrical field stimulation

Next, we analysed whether miR‐146b‐5p transfection (30 nmol/L, Figure S6) in hiPSC‐aCMs affected the expression of TIMP‐4, MMP9, TGF‐β1, COL‐I and the interstitial collagen deposition in the co‐cultured cellular model by RES. After transfection of miR‐146b‐5p in hiPSC‐aCMs, TIMP4 mRNA expression level was significantly decreased (*p* < 0.001), while MMP9, TGFB1 and COL1A1 expression was significantly increased without RES (*p* < 0.05). Moreover, after 24 h, rapid depolarization of hiPSC‐aCMs by RES, MMP9, TGFB1 and COL1A1 mRNA expression was all significantly increased compared with those before RES (*p* < 0.05, Figure 4A). Meanwhile, these mRNA expression changes were associated with corresponding protein levels detected by Western blot analysis (Figure 4B). Although the possibility of RES changing the mentioned mRNA expression in the fibroblasts (independent of the hiPSC‐aCMs) remains optimistic, our results revealed the remained mRNA expression in the fibroblast‐only culture, even with the rapid pacing. (Figure S7).

We also observed the ultrastructure of hiPSC‐aCMs‐fibroblast by transmission electron microscopy (TEM). The experimental results revealed that collagen fibres were accumulated in extracellular space after rapid depolarization of miR‐146b‐5p overexpressed hiPSC‐aCMs‐fibroblast co‐culture cellular model by RES (Figure 4C).

To sum up, phenotype change in miR‐146b‐5p transfected hiPSC‐aCMs‐fibroblast co‐culture cellular model by RES suggested that the interaction mechanism of electrical and structural remodelling has a crucial role in AF.

### 3.4 Inhibiting miR‐146b‐5p reduces cardiac fibrosis after MI in mice

**FIGURE 5:**
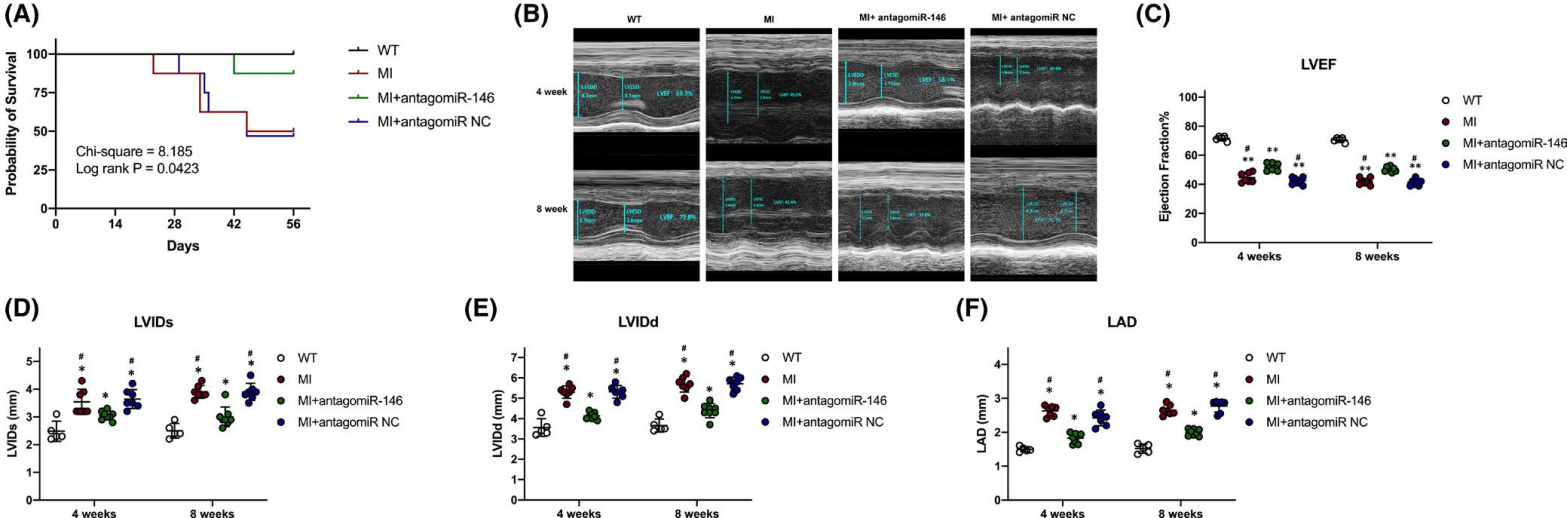
A, Comparison Kaplan‐Meier survival curves after MI in mice during an 8‐week follow‐up period. B, Representative M‐mode echocardiograms of a mouse from a different group at baseline, 4 weeks and 8 weeks after MI. (C) The 4‐ and 8‐week echocardiographic data for ejection fraction (EF), (D) LVID at systole (LVIDs), (E) LVID at diastole (LVIDd) and (F) LAD. G, Representative surface ECG pacing‐induced AF in mice. (H) AF inducibility increased significantly in in both MI mice and antagomiR‐NC pretreated MI mice. ^*^ *p* < 0.05 versus WT; ^**^ *p* < 0.001 versus WT; ^#^ *p* < 0.05 versus MI+antagomir‐146. LAD, left atrial diameter; LVID, left ventricular internal diameter; MI, myocardial infarction; NC, negative control; WT, wild type

Based on the previous discovery, our goal became to examine whether inhibiting the expression of miR‐146b‐5p reduces the phenotypes of cardiac fibrosis in the MI mouse model. The mice were subjected to experimental MI and pretreatment with injecting antagomiR‐146b‐5p by tail vein to knock down the endogenous origin of miR‐146b‐5p for 3 days. We found that antagomiR‐146b‐5p significantly improved post‐MI survival during 8 weeks after MI (Figure 5A). Echocardiography was performed at 4 and 8 weeks after MI to assess cardiac function (Figure 5B). Within expectation, ejection fraction (EF) decreased in all groups of mice after MI (*p *< 0.001). However, antagomiR‐146b‐5p mice demonstrated a significantly preserved EF than those two group mice at 4 and 8 weeks after MI (*p *< 0.05) (Figure 5C). The improved EF in antagomiR‐146b‐5p mice after MI was the culmination of substantially improved cardiac function, as evidenced by the significantly smaller LV internal diameter measured at systole and diastole (*p *< 0.05) (Figure 5D‐E). Meanwhile, although the LA size was increased in antagomiR‐146b‐5p treated mice after MI, it was smaller than those two groups of mice after MI (*p *< 0.05) (Figure 5F). No spontaneous AF was observed. However, a typical disorganized atrial tachycardia induced by programmed electrical atrial pacing after carbachol injection was easily and reproducibly inducible in both MI mice (19 of 22) and antagomiR‐NC pretreated MI mice (18 of 22), compared with that in WT mice (4 of 20) and antagomiR‐146b‐5p pretreated MI mice (10 of 23) (Figure 5G–H).

**FIGURE 6:**
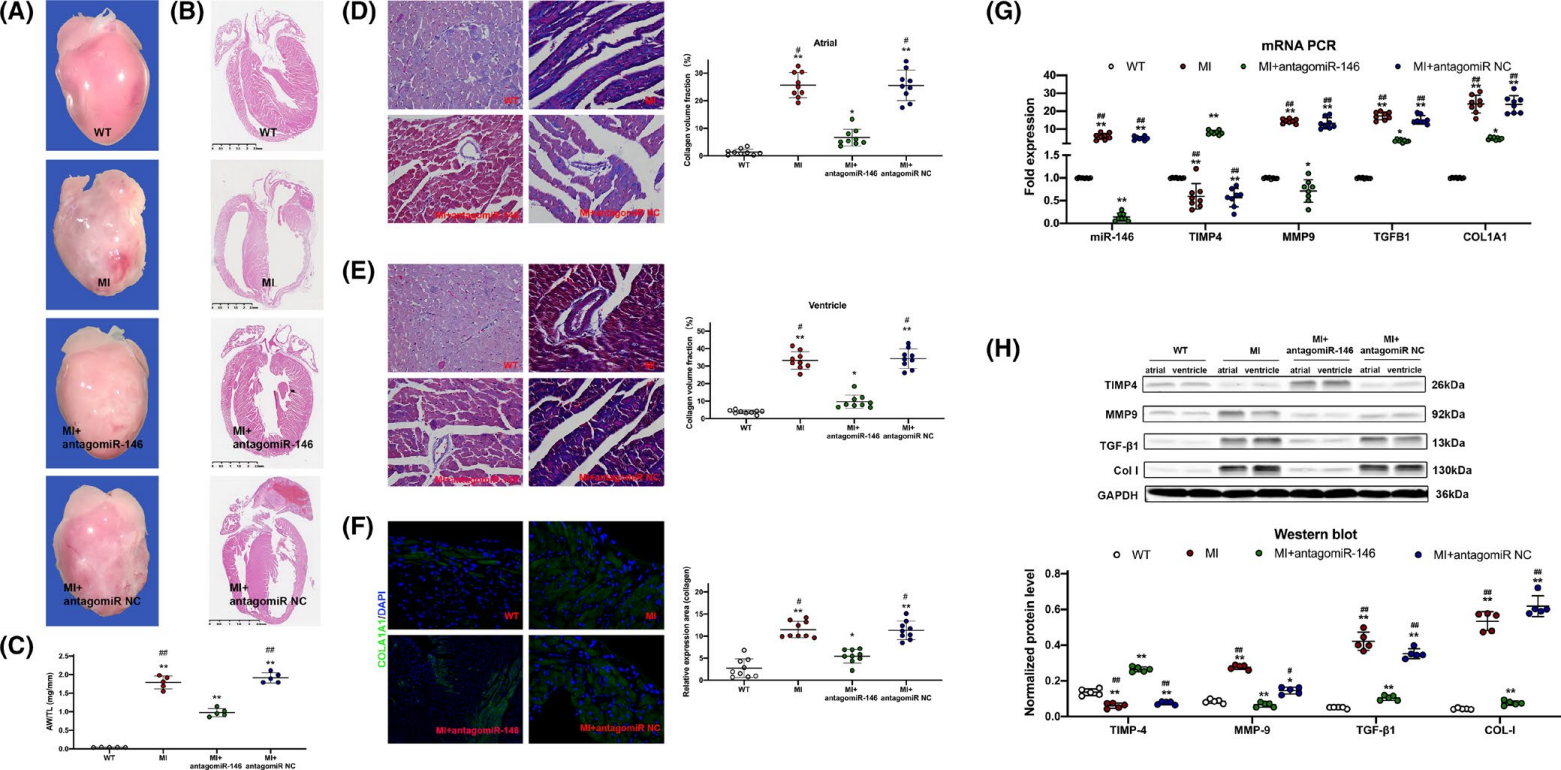
A, Examples of whole hearts showing evidence of atrial dilatation in mice after MI. B, Whole heart long‐axis images of haematoxylin‐eosin staining. C, Atrial weight/tibia length (AW/TL) ratios of WT and other 3 groups mice 8 weeks after MI. D‐E, Representative Masson's trichrome images and quantification of atrial (D), and ventricle (E) fibrosis after 8 weeks of MI compared with WT. Quantification expressed as collagen volume fraction. F, Representative immunofluorescence images showing COL1A1 (green) and DAPI (blue) in WT and other 3 groups hearts 8 weeks after MI. G, PCR analysis showed mRNA expression of miR‐146b‐5p, MMP9, TGFB1and COL1A1. H, Western blot analysis showed protein expression of TIMP4, MMP9, TGF‐β1 and COL‐I in cardiomyocytes. ^*^ *p* < 0.05 versus WT; ^**^ *p* < 0.001 versus WT; ^#^ *p* < 0.05 versus MI+antagomir‐146; ^##^ *p* < 0.001 versus MI+antagomir‐146. MI, myocardial infarction; NC, negative control; WT, wild type

There was a clear biatrial enlargement in mice after MI 8 weeks compared with WT mice (Figure 6A,B). Quantification of the atrial weight normalized to tibia length revealed that the atrial mass was significantly increased in all mice after MI compared with WT mice. However, after applying antagomiR‐146b‐5p after MI, the atrial mass was significantly reduced (Figure 6C).

In order to determine the fibrotic response, we utilized Masson's trichrome staining to stain fibrotic areas within the myocardium (Figure 6D‐E). All mice after MI showcased a fibrosis increase in the LA and LV, while antagomiR‐146b‐5p treated MI mice only had a significant increase in LV fibrosis. Consistent with these results, the collagen volume fraction was significantly reduced in antagomiR‐146 treated mice after MI in both regions. Immunofluorescence also showed that antagomiR‐146 decreased in the expression of collagen‐1 protein compared with the other 2 groups of mice after MI (Figure 6F). Fibrotic marker MMP9, TGFB1 and COL1A1 were significantly downregulated, while TIMP4 was significantly upregulated at both mRNA and protein levels by miR‐146b‐5p inhibition in CMs of MI heart (Figure 6G‐H). These results suggest that the loss of miR‐146b‐5p can protect against MI‐mediated cardiac fibrosis.

## 4 DISCUSSION

**FIGURE 7:**
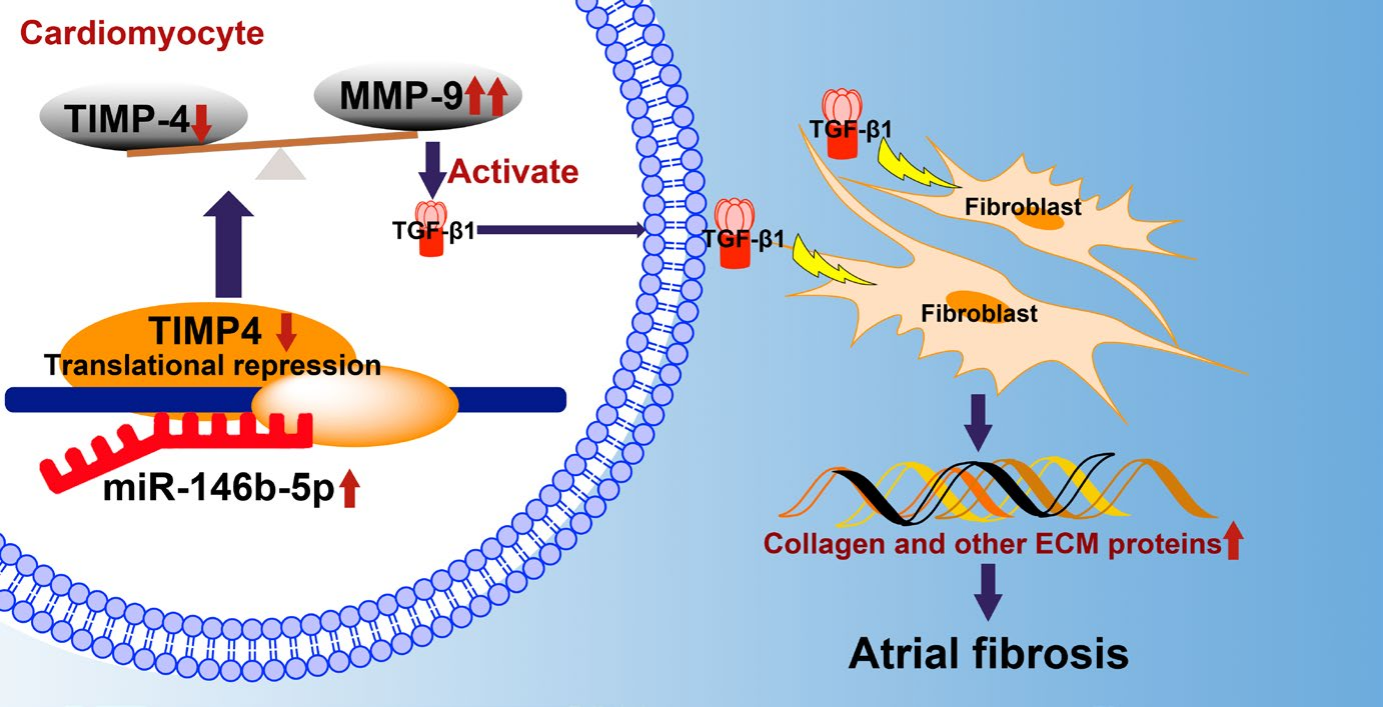
Proposed working model of the miR‐146b‐5p mediated atrial fibrosis in atrial fibrillation

This research discussed the function of miR‐146b‐5p in hiPSC‐aCMs‐fibroblast co‐culture cellular model *in vitro* and murine heart *in vivo*. miR‐146b‐5p has a critical role in the regulation of atrial fibrosis, contributing to the structural remodelling in AF. It demonstrated that collagen fibres were accumulated in extracellular space after RES on miR‐146b‐5p overexpressed co‐culture cellular model. Moreover, the cardiac fibrosis induced by MI was reduced in antagomiR‐146 treated mice, indicating that the inhibition of miR‐146b‐5p might become an effective therapeutic approach to prevent atrial fibrosis. To our understanding, this will be the initial study that investigated the unknown mechanism of miR‐146b‐5p on atrial fibrosis in AF. Our proposed working model of the miR‐146b‐5p mediated atrial fibrosis in AF is shown in Figure 7.

TIMP4 is endogenously one of the key modulators of MMP9, which is activated in atrial fibrosis.^18^The activation of MMP9 beyond a certain limit is deleterious to the heart, which worsens cardiac remodelling.^5^, ^19^, ^20^ The hyperearly research discovered that when the expression of miR‐146b‐5p increases, TIMP4 is down‐regulated due to repression in cardiomyocytes in patients with AF.^15^ In the present study, we further evaluated miR‐146b‐5p and TIMP4 in ATP canine and observed a significant negative correlation between them. As a result, there is a shift in the TIMP4‐MMP9 axis towards the MMP9, which has been reported to activate latent TGF‐β1 to its active form and to induce TGF‐β1 production, which interacts with neighbouring fibroblasts and promote collagen synthesis, as well as cardiac fibrosis.^21^

In patients with AF, electrical and structural remodelling normally comes together; thus, their effects are difficult to separate. ATP animal models typically develop some degree of structural remodelling.^22^ The highly homogeneous aCMs with normal electrophysiological properties, generated from hiPSC, stand for a promising and powerful method for screening anti‐arrhythmic drugs *in vitro* on a wide spectrum. Studies have shown that tachy‐paced aCMs promotes protein synthesis by atrial fibroblasts.^23^ Thus, we used a direct hiPSC‐aCMs‐fibroblast contact co‐culture system to investigate the possible molecular mechanism of atrial fibrosis.

After using RES, we found that the APD of hiPSC‐aCMs was markedly shortened with a marked reduction in *I_Ca_*_,_ *_L_*, which was consistent with the atrial electrical remodelling process during AF. Yang et al^24^ demonstrated that aCMs subjected to RES in culture undergo electrical remodelling (shorten APD and reduction in *I_Ca_*_,_ *_L_*), preserving principal phenotypic features of remodelling *in vivo*, which is consistent with our results. RES of cardiomyocytes causes repetitive entry of Ca^2+^ into the cell through sarcolemmal channels, with progressive intracellular Ca^2+^ loading. Consequently, the downregulation of *I_Ca_*_,_ *_L_* occurs as an adaptive cellular response to minimize Ca^2+^ overload. Although Tsai et al reported RES of aCMs induced expression of COL1A1 in co‐cultured atrial fibroblasts, no changes of the expression on MMP9, TGFB1 and COL1A1 were found after RES in our study.^23^ After transfection of miR‐146b‐5p in hiPSC‐aCMs, TIMP4 mRNA expression level was significantly decreased, while MMP9, TGFB1 and COL1A1 expression were significantly increased without RES. Moreover, after using RES, TGFB1 and COL1A1, mRNA expression was most significantly increased compared with those before RES. These results showed that our co‐culture cellular model maximum mimicked the electrical and structural remodelling in AF. Also, phenotype change in miR‐146b‐5p transfected co‐culture cellular model after RES implicated overexpressed miR‐146b‐5p in hiPSC‐aCMs may cause structural remodelling by affecting fibroblast function and may contribute to the upregulation of fibrotic marker in response to RES.

Finally, we decided to examine whether the inhibition of miR‐146b‐5p may attenuate the phenotypes of atrial fibrosis in the MI mouse model. In this study, mice treated with antagomiR‐146b‐5p showed better survival rate and cardiac function, which could be interrupted by less cardiac fibrosis after MI. Consistent with these results, the fibrotic marker MMP9, TGFB1 and COL1A1 were significantly downregulated, while TIMP4 was significantly upregulated at both mRNA and protein levels by miR‐146b‐5p inhibition in the MI heart.

Studies have shown that several miRNAs, including miR‐21, miR‐24, miR‐29 and miR‐146, are related to cardiac fibrosis.^25^, ^26^, ^27^ Most previous studies have focussed on miR‐21, which is involved in pathophysiological cardiac fibrosis via different signalling pathways. Contrary to our results, Di et al found that the infarct size in the ischaemia/reperfusion model was reduced and the ischaemia/reperfusion‐induced H9C2 apoptosis *in vitro* was attenuated by the miR‐146b overexpression.^28^ However, Kazimierczykin et al discovered that levels of plasma miR‐146 significantly increased as compared to the healthy volunteers in the early phase of MI, while significantly decreased in the post‐healing phase.^29^ Furthermore, in a recent study, Liao et al stated the upregulated miR‐146b‐5p in the infarcted myocardium of mice as well as the serum of myocardial ischaemia patients.^14^ Local administration of a miR‐146b‐5p antagomir greatly decreased fibrosis in the infarcted myocardium to restore cardiac remodelling and function in MI models. They found that the above phenotypic modulations included interleukin 1 receptor associated kinase 1 (IRAK1) and carcinoembryonic antigen related cell adhesion molecule 1 (CEACAM1), which did not change in our study, was mediated by the miR‐146b‐5p target genes (Figure S8). This research discovered that miR‐146b‐5p repressed target gene TIMP4, which can be cardioprotective when overexpressed in cardiac tissues and can modify extracellular matrix remodelling. These differential target genes may be explained by our use of aCMs of patients with AF rather than serum of myocardial ischaemia patients.

Taken together, the aforementioned evidence raised the possibility of potentially exploiting miR‐146b‐5p as a novel target to develop effective therapeutic strategies for cardiovascular diseases in the near future. Further extensive studies from bench to bedside are required to reveal the clinical and mechanistic roles of miR‐146b‐5p during the process of AF associated with atrial fibrosis and other cardiovascular diseases.

This study has certain limitations. First, although hiPSC‐aCMs‐fibroblast co‐culture was an AF cellular model, specific inherent restriction generating from the hiPSC‐aCM model will be needing further study. Specifically, the problem with immaturity remains critical. Second, this research primarily studied the miR‐146b‐5p regulation of TIMP4 proteins, since the importance of the TIMP4/MMP9 axis in the predominance of structural remodelling changes. However, the differences in the miR‐146b‐5p level possibly lead to arrhythmogenesis by adjusting restructuring in cardiomyocytes. This is an area that merits further exploration.

## 5 CONCLUSIONS

Our results demonstrated that the overexpression of miR‐146b‐5p activates collagen synthesis and deposition in an AF cellular model, while its inhibition could attenuate the atrial fibrosis and preserve heart function after MI in the mouse heart. This study was the first to demonstrate a mechanical link between miR‐146b‐5p and atrial fibrosis in AF. Our findings also indicated that the inhibition of miR‐146b‐5p hinders the TIMP4/MMP9 unbalance related restructuring in AF and might compose a new anti‐AF method aiming atrial fibrosis.

## CONFLICT OF INTEREST

The authors declare that they have no competing interests.

## AUTHOR CONTRIBUTION

Qing Ye: Data curation (equal); Investigation (equal); Methodology (equal); Writing‐original draft (equal). Quan Liu: Data curation (equal); Investigation (equal); Methodology (equal); Validation (equal); Writing‐original draft (equal). Xiaolong Ma: Data curation (equal); Formal analysis (equal); Investigation (equal); Methodology (equal); Writing‐original draft (equal). Shuyun Bai: Investigation (equal); Methodology (equal); Writing‐original draft (equal). Pengfei Chen: Investigation (equal); Methodology (equal). Yichen Zhao: Data curation (equal); Methodology (equal). Chen Bai: Data curation (equal); Methodology (equal). Yang Liu: Data curation (equal). Kemin Liu: Formal analysis (equal). Meng Xin: Investigation (equal). Caiwu Zeng: Data curation (equal). Cheng Zhao: Data curation (equal). Yan Yao: Supervision (equal). Yue Ma: Supervision (equal); Writing‐original draft (equal). Jiangang Wang: Conceptualization (lead); Data curation (lead); Formal analysis (lead); Funding acquisition (lead); Investigation (lead); Methodology (lead); Project administration (lead); Supervision (lead); Writing‐original draft (lead); Writing‐review & editing (lead).

## Supporting information

Table S1

Table S2

Supplementary Material

Supplementary Material

## Data Availability

The data that support the findings of this study are available from the corresponding author upon reasonable request.
