# Supplementary material for: MicroRNA‐146b‐5p promotes atrial fibrosis in atrial fibrillation by repressing TIMP4
Source: J Cell Mol Med. 2021 Oct 13;25(22):10543–53. doi: 10.1111/jcmm.16985 (PMC8581305; doi:10.1111/jcmm.16985)
Supplement: Supplementary file 3 — Supplementary Material [file JCMM-25-10543-s004.docx]

SUPPLEMENTAL MATERIAL.

**Methods**

***HiPSC derived atria-like cardiomyocytes***

In a previous study, highly homogenous atrial-like cardiomyocytes (aCMs) were obtained from human pluripotent stem cells. The hiPSC-aCMs from Cauliscell Company (Cat# 300206) were cultured following the manufacturer’s protocols. Briefly, after thawing, 1.5–2 × 10^6^ hiPSC-aCMs were plated onto six-well plates with 2 mL of cardiomyocytes plating medium. Twenty-four hours after thawing, the medium was replaced with cardiomyocyte maintenance medium and changed every 2 days.

***hiPSC-aCMs–fibroblast contact co-culture and rapid electrical field stimulation***

The fibroblasts were trypsinized and seeded onto the culture dish, in which the hiPSC-aCMs had been cultured in medium to 50–70% confluence and were in spontaneous synchronized contraction. Twenty-four hours later the hiPSC-aCMs and fibroblasts were in the confluent phase and grew in monolayers, and the hiPSC-aCMs were still in spontaneous synchronized contraction. Then the culture medium was changed to serum-free Tyrode solution. The Tyrode solution contained: NaCl, 137 mM; KCl, 5.4 mM; MgSO_4_, 1.2 mM; CaCl_2_, 1.8 mM, KH_2_PO_4_, 1.2 mM, dextrose, 22 mM; and, 12 mM (pH 7.4). After 6 h of serum-free culture, the cells were subjected to rapid field pacing in an incubator containing an atmosphere of air enriched with 5% CO2 at 37°C for the indicated times.

Two parallel copper panel spaced 1 cm apart from the 3.5 cm culture dish were placed. The two panel were connected to the anode and cathode of a YC-2-S stimulator with a biphasic square waveform. The stimulator delivered 1.5 volt to the panels, creating a voltage gradient of 1.5 V/cm.RES was applied at the frequency of 10Hz.

***Study animals***

Animal protocols were approved by the Institutional Animal Care and Use Committee of the Capital Medical University (20170015), and performed in accordance with the guidelines set forth in the Guide for the Care and Use of Laboratory

All mice used in this study were on the C57BL/6 background. Mice were group-housed under a 12:12-hour light: dark cycle at 25°C and had unrestricted access to food and water. Female mice (age 6-8 weeks) were used for experiments.

Beagle dogs were housed individually in stainless steel cages from the time of arrival until euthanasia. Beagle dogs were separately housed at cages at a temperature of 13‑16˚C and humidity of 40‑70%, under 12‑h light/dark cycles. Sufficient drinking water was provided for each Beagle dog, and high‑quality adult dog food (3‑5% of its weight) was provided twice a day.

***IECD system***

The implantable electronic cardiovascular devices (IECDs) system was provided by Genix Biotek Science Technology (Shanghai) Co., Ltd. (Shanghai, China). The IECD consists of one electronic component box, <5 ml in volume and 15 g in weight, two ECG monitoring electrodes and one bipolar stimulation electrode (5076 lead; Medtronic, Inc., Minneapolis, MN, USA). The extracorporeal system consisted of a signal transmit-receive box which connected with the internet or the third-generation communication system (virtual private network system), and a laptop with ECG monitoring and electrical stimulation software. The laptop was also used as a central workstation in the system. The laptop received remote digital signals and converted them into real-time ECG data. The real-time ECG was saved as a compressed ECG signal file with precise time stamps for off‑line analysis. When the IECD was implanted, the extracorporeal system also remotely sent out a stimulation signal wirelessly to the IECD to stimulate the atrium via stimulation electrodes.

The regular interval stimuli modes (S1S1) in this IECD system were used in this study. The stimulation parameters of this IECD system were as follows: stimulation current between 2 mA, stimulation time length 3 msec, interval 4 ms, and stimulation rate 30 Hz.

***Canine model of AF***

Twelve healthy, male Beagles, weighing between 8 and 12 kg, were randomly divided into two groups: sham control (n=6) and atrial tachypacing (ATP, n=6) group. All were anesthetized with ketamine (1 mg/kg; Jiangsu Hengrui medical Co., Ltd', Jiangsu, China; batch no. 20101105). Organon Vecuronium (0.1 mg/kg; Merck & Co., Inc., Whitehouse Station, NJ, USA; batch no. 403138) was used to inhibit spontaneous breathing. Tracheal intubation cannula was used to assist with respiration by anesthesia machine (Excel 210, Ohmeda Anesthesia System, BOC Health Care, USA), and Propofol (200 µg/kg/min; Xi'an Libang Pharmaceutical Co, Ltd., Xi'an, China; batch no. 0711192) was used to maintain anesthesia.

Anesthetized dogs were ventilated mechanically with room air at a rate of 8 to 12 breaths per minute and a tidal volume of approximately 20 ml/kg, thus sustaining the arterial partial pressure of CO_2_ between 35 to 45mm Hg, that of O_2_ greater than 80 mm Hg, and pH at 7.38 to 7.45. Body temperature was maintained between 37.0 and 37.5 °C.

All canines were anesthetized, and the left dorsal skin was incised to implant an IECDs, and a pair of pacemaker electrodes was sutured subcutaneously. The dog left atrial appendage (LAA) were exposed through the right fourth intercostal space, a pair of pacemaker electrodes was sutured on the LAA to induce AF and the distance between two electrodes was about 10 mm. After the operation, the monitor system was started and continuously monitored the ECG signal. Penicillin sodium (800,000 IU) was injected intramuscularly every day to fight infection for 7 days. Warfarin was administration 5 days later and the stimulation system was started 7 days later in high-frequency stimulation pattern (stimulate duration 3 s, interval 4 s, amplitude 2mA at 30Hz). The dogs were subjected to left atrial pacing for 8 weeks before experimental studies. Sham-operated canine received the same procedures as the experimental canines but without implant of pacemaker electrodes. Atrial fibrillation was identified when (1) the ventricular response was rapid and irregularly irregular, (2) P waves were absent, (3) low-frequency, irregular oscillations (f waves) were present, and (4) systemic arterial pressure pulses occurred irregularly, were variable in amplitude, and accompanied a pulse deficit (that is, fewer pulsations than QRS complexes occurred).[9](https://www.ncbi.nlm.nih.gov/pmc/articles/PMC2707127/#bib9) The time between cessation of atrial tachy-pacing and return to SR was measured.

***MI in wild-type mice***

MI was induced in 10-week-old wild-type (WT) C57BL/6 mice by permanent ligation of the left anterior descending coronary artery (LAD). Briefly, the mice were doped with anesthesia (10% chloral hydrate, 0.3 mL/100 g, i.p.) and maintained under artificial ventilation. The chest cavity was opened, and after careful dissection of the pericardium, LAD was permanently ligated using a 7-0 silk suture.

***Effect of different miRNA concentrations on exogenous TIMP4***

To confirm that miR-146b-5p suppress TIMP4 translation, we transfected human fibroblasts with an expression plasmid containing the full-length TIMP4 mRNA driven by a CMV promoter. When fibroblasts were transfected with varying amounts of miR-146b-5p mimics, we noted maximal suppression of TIMP4 with 30 nmol/L miR-155.

***Lentivirus packaging and transfection***

MiR-146b-5p agomir or antagomir and control RNA duplex (NC) were synthesized by RiboBio (Guangzou, China). HEK-293T cells (~70% confluent) were transfected with 100nM miRNA agomir, antagomir, siRNA, or NC using LipofectamineRNAiMAX (Invitrogen) following the manufacturer's instructions. The lentivirus (GenePharma) was used to construct stable cell lines transfected with miR-146b-5p mimics or inhibitors using a Lenti-Pac HIV Expression Packaging Kit (GeneCopoeia). The cells without miR-146b-5p mimic or inhibitor transfection was used as an NC. The supernatant of HEK-293T cell culture was then condensed to a viral concentration of approximately 3 × 10^8^ transducing units/mL. For transfection, the lentiviral particles were incubated with hiPSC-aCMs for 8 h. The stably transfected cells were selected using green fluorescent protein.

***Whole-cell patch-clamp recording***

Patch-clamp techniques were applied to isolated atrial myocytes from hiPSC-aCMs and mice. Briefly, the pipette had the tip resistance of 2–3 MΩ when filled with pipette solution. The isolated single cells were placed in a 1-mL chamber mounted on an inverted microscope (IX-70, Olympus) and perfused with Tyrode solution. Whole-cell recording were performed using an amplifier (EPC-10, HEKA, Germany). Ion currents were recorded in the whole-cell voltage-clamp mode. For the recording of L-type Ca^2+^ current (*I_CaL_*), the pipette solution contained the following (in mM): CsCl, 120; MgCl_2_; 1; HEPES, 10; EGTA, 10; Na2-GTP 0.3; and Mg-ATP (pH 7.2 with CsOH), 4. The external solution contained the following (in mM): TEA-Cl, 140; 2 MgCl_2_ ⋅ 6H_2_O, 2; CaCl_2_, 10; HEPES, 10; and D-glucose (pH=7.4 with TEA-OH), 5. For recording inward rectifier K^+^ current (*I_K1_*), transient outward K^+^ current (*I_to_*), and ultrarapid delayed rectifier K^+^ current (*IK_ur_*), the pipette solution contained the following (in mM): KCl, 20; K-Aspartic, 115; MgCl_2_, 1; EGTA, 5; HEPES, 10; and Na2-ATP (pH 7.2 with KOH), 2. The external Tyrode solution contained the following (in mM): NaCl 140; KCl, 3.5; MgCl_2_ ⋅ 6H_2_O, 1; CaCl_2_, 2; D-glucose, 10; HEPES, 10; and NaH_2_PO_4_ (pH 7.4 with NaOH), 1.25. Nifedipine (10μM) was included to inhibit *I_CaL_*. For recording Na current (*I_Na_*), the pipette solution contained 50mM CsCl, 10mM NaCl, 20mM EGTA, 10mM HEPES, and 60mM CsF, pH 7.2 with CsOH.

Experiments were conducted at 36 ± 1°C. Junction potentials were zeroed before the formation of the membrane–pipette seal and not corrected for data analyses. Series resistance and capacitance were compensated. No leak current subtraction was made. Cells with significant leak currents were rejected. For analysis, the data were recorded on an IBM-compatible computer and analyzed with the use of Igor Pro 6.0 software. *I_Ca,L_* was elicited using 300-ms depolarizing pulses delivered from a holding potential of –60 mV at a frequency of 0.1 Hz. *I_to_* and *I_Kur_* were evoked by 1000-ms depolarizing pulses ranging from –40 mV to +50 mV from a holding potential of –50 mV at a frequency of 0.1 Hz. *I_to_* was measured as the difference between the peak current amplitude and the sustained current level, and *I_Kur_* was defined as the current amplitude at the end of the 1000-ms pulse. For all recordings, sodium current was inactivated by the holding potentials at a voltage of –50 mV or higher. *I_Na_* was recorded from a holding potential at –90mV and then elicited using 50-ms depolarizing pulses from –120 mV to 100 mV with an increment of 5 mV.

This study was designed for group comparisons of the experimental results. Hence, all the currents were recorded immediately after membrane rupture and series resistance compensation to minimize the possible time-dependent rundown of 14 currents. Individual currents were normalized to the membrane capacity to control for the differences in cell size, which was expressed as current density p*A*/p*F*. Single-cell action potentials were recorded under the current-clamp mode, and a stimulatory current sufficient to induce action potential was used in this experiment. The action potential duration for both 50% and 90% repolarization (APD50 and APD90) was analyzed.

***Echocardiography***

Transthoracic echocardiographic analyses were undertaken on lightly anesthetized mice by using a Vevo 2100 imaging system (Visualsonics, Toronto, Canada) equipped with a 40-MHz RMV 704 probe. Mice were imaged on a heated table with surface ECG recordings after intraperitoneal anesthesia with 1.25% avertin. If heart rate fell below 450 beats per minute the session was re-performed. Two-dimensional guided M-mode tracings were recorded at the level of the papillary muscles in the ventricular short axis show for the measurements of ventricular parameters, including systolic left ventricular internal diameters (LVIDs), diastolic left ventricular internal diameters (LVIDd) and left ventricular ejection fraction (LVEF). Left atrial size was measured on the basis of its antero-posterior diameter on parasternal long-axis view at end systole. Continuous-wave Doppler was used for measurements of E peak velocity, A peak velocity and E/A ratio at apical four chambers view. All above measurements were made from >3 beats and the results averaged. Echocardiographic parameters were obtained after cardiac function had been evaluated.

***Masson's staining***

The heart tissue sample was fixed in 10% buffered formalin and embedded in paraffin. Sections at 4 µm were stained with Masson's staining. The collagen volume fraction was calculated as the ratio of the total area of interstitial fibrosis to the entire visual field of the section. A minimum of 5 randomly selected areas per sample were observed at ×400 magnification, and the average value was calculated for statistical analysis.

***Transmission electron microscopy***

hiPSC-aCMs prior to and following RES were collected, transferred into Eppendorf tubes, resuspended in cold PBS and centrifuged at 200 x g for 5 min at 4 ̊C. The supernatant was removed and cells were fixed in 2% glutaraldehyde for 2 h and post‐fixed in 1% tetroxide osmium for 2 h. Following dehydration with an alcohol gradient, cells were embedded in epoxy resin 618 (Shanghai Kang Lang Biological Technology Co., Ltd., Shanghai, China). Ultrathin sections (100 nm) were prepared and contrast stained with uranyl acetate and lead citrate. Images were captured (magnification, x6,000) using a transmission electron microscope (H7700; Hitachi, Ltd., Tokyo, Japan).

***Mice transfected with adeno-associated virus miR-146b-5p inhibitor***

A cardiac-specific adeno-associated virus (AAV) vector carrying miR-146b-5p inhibitor, was generated in Hanbio. Transfection of AAV-miR-146b-5p inhibitor 50 µl (1×10^11^PFU) was injected via tail vein for 7 days.

***Quantitative real-Time polymerase chain reaction***

The reverse transcription of RNA was conducted to measure the level of miRNAs using a TaqMan microRNA Reverse Transcription kit (ABI) following the manufacturer’s recommendations. Subsequently, 3 μL of the product was used for detecting microRNA (miRNA) expression by quantitative real-time polymerase chain reaction (RT-PCR) using TaqMan microRNA Assay kits (ABI) for the corresponding miRNA. For the mRNA, the total RNA was reverse transcribed using M-MLV Reverse Transcriptase (Invitrogen) and random primers. Then, the product was used for detecting mRNA expression by quantitative RT-PCR using Power SYBR Green PCR Master Mix (ABI) for the corresponding mRNA. The PCR was conducted using a 7900HT Sequence Detection System (Applied Biosystems, TX, USA). All of the reactions were run in triplicate. The values of the different miRNAs were normalized to cel-miR-39. The relative expression levels of the mRNAs were calculated based on GAPDH levels and multiplied by 10^2^.

***Western blot analysis***

Human induced pluripotent stem cell derived atrial cardiomyocytes (hiPSC-aCMs) or tissues were homogenized in RIPA buffer supplemented with protease and phosphatase inhibitors (ThermoFisher Scientific). Equal amounts of cell lysate proteins from each biological replicate were subjected to Western blot analysis. Using a ChemiDoc XRS imaging system (BioRad), the protein bands on blots were detected with a SuperSignal West Pico Chemiluminescent Substrate (Thermo Scientific). Protein bands were analyzed using Image Lab software (BioRad). Arbitrary densitometry units were quantified and expressed as mean ± SEM. The phosphorylated proteins were normalized to total protein bands, or protein expression was normalized to housekeeping protein bands. Western blot data in figures and supplemental figures were all representatives of more than three independent experiments.

***Statistical analysis***

All results for continuous variables were expressed as mean ± standard deviation of the mean, and categorical variables were expressed as the number of patients and percentage. The significance of the differences between the groups was assessed using the Student *t* test or one-way analysis of variance for continuous variables and the *χ*^2^ test for categorical variables. Logistic regression analyses were performed to identify the relationship between the variables. All tests were two tailed, and a *P* value of 0.05 was considered to indicate the significance. The data were analyzed in Igor Pro 6.0 (WaveMetrics, Inc. OR, USA) or GraphPad Prism 8.0 (GraphPad Software Inc, CA, USA).

**
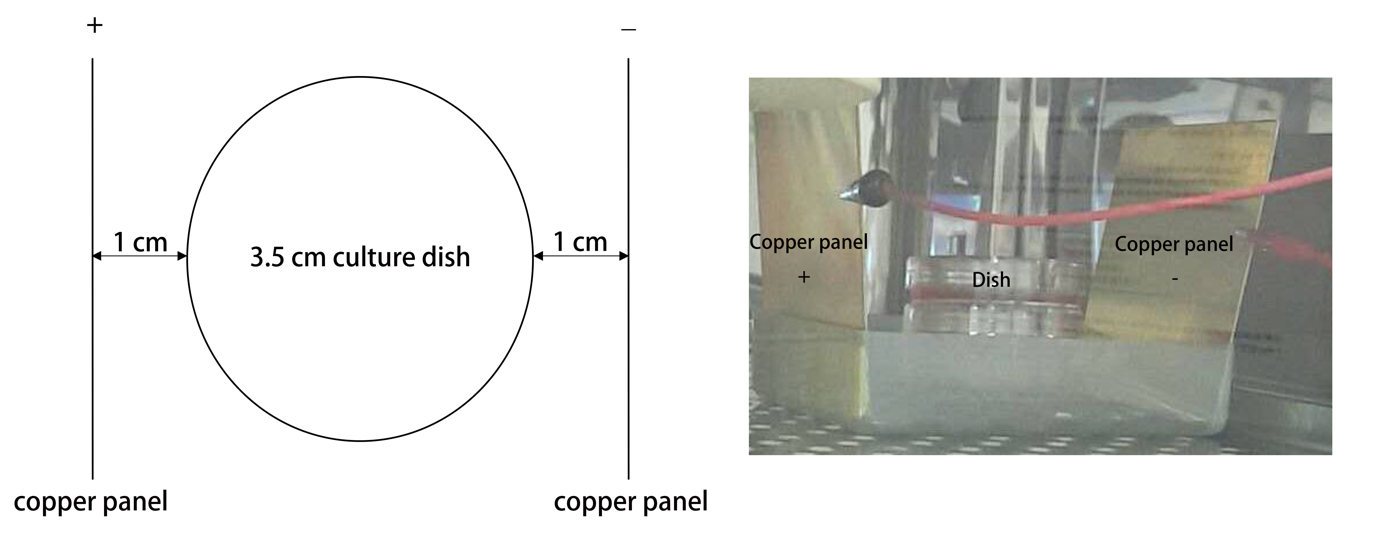
**

**Supplemental Figure 1** Representative of rapid electrical field stimulation. Two parallel copper panel spaced 1 cm apart from the 3.5 cm culture dish were placed.


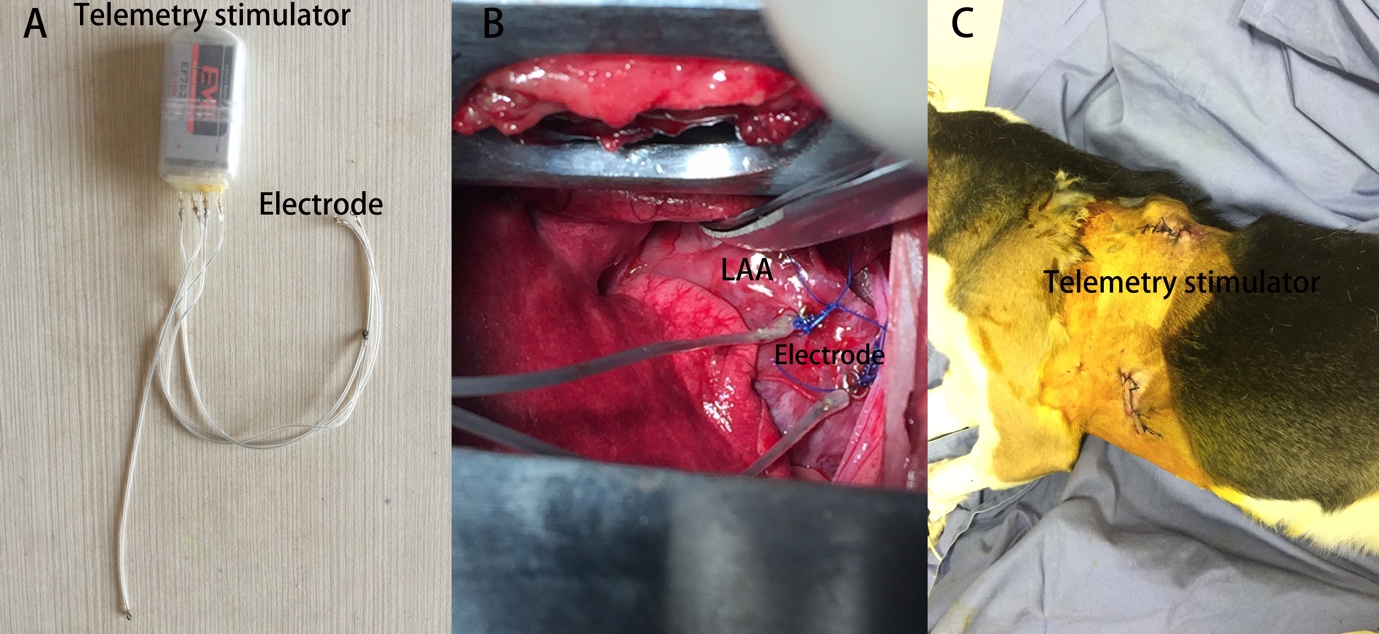


**Supplemental Figure 2** Canine model of atial fibrillation by atrial tachypacing. **(A)** A implantable IECDs, and a pair of pacemaker electrodes. **(B)** The dog left atrial appendage (LAA) were exposed through the right fourth intercostal space, a pair of pacemaker electrodes was sutured on the LAA to induce AF and the distance between two electrodes was about 10 mm. **(C)** All canines were sucsussfully implanted an IECDs under left dorsal skin.


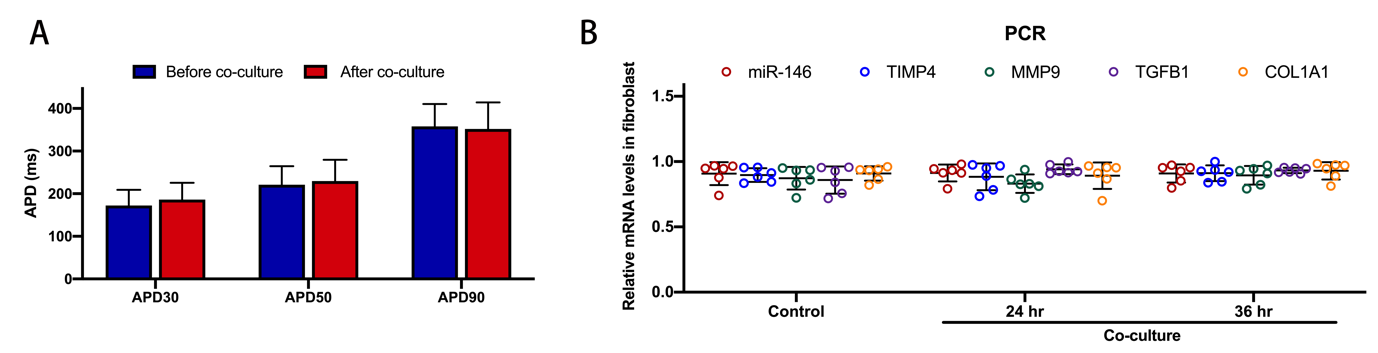


**Supplemental Figure 3** Electrophysiological properties in hiPSC-aCMs **(A)** and fibrosis related mRNA expression in the fibroblasts **(B)** after hiPSC-aCMs-fibroblast contact co-culture. APD, action potential duration.


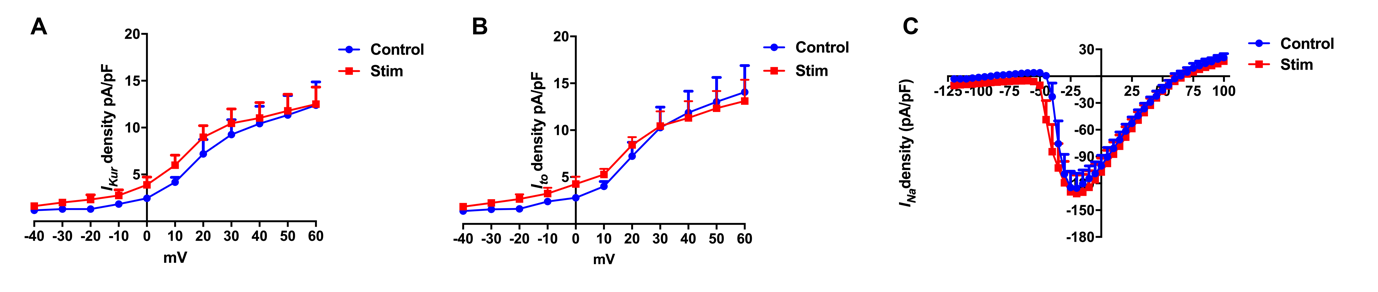


**Supplemental Figure 4 A**fter rapid electrical field stimulation there was no change on the current densities of *I_to_*, *I_Kur_*, and *I_Na_* in hiPSC-aCMs.


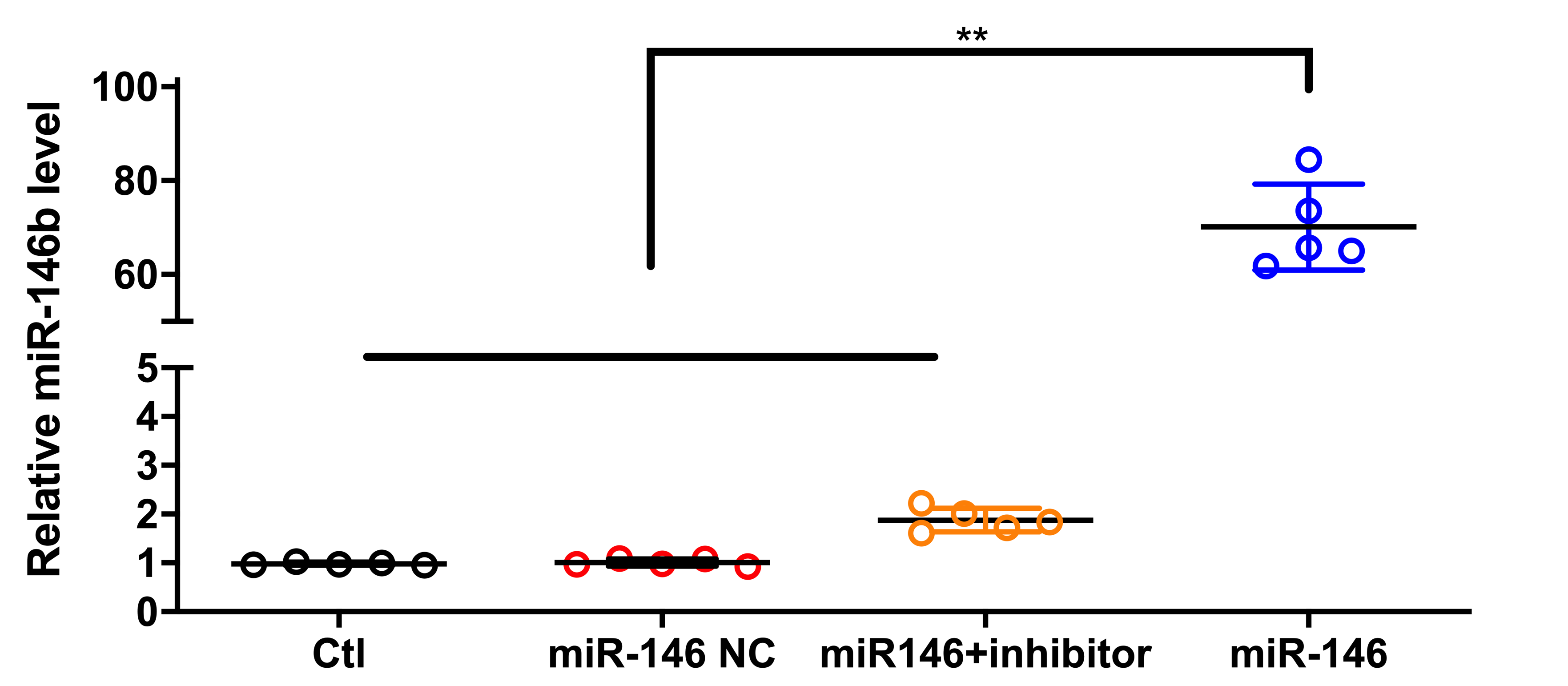


**Supplemental Figure 5** PCR results revealed that the miR-146b-5p inhibitor could significantly reduce the overexpression of miR-146b-5p in cardiomocytes.

**
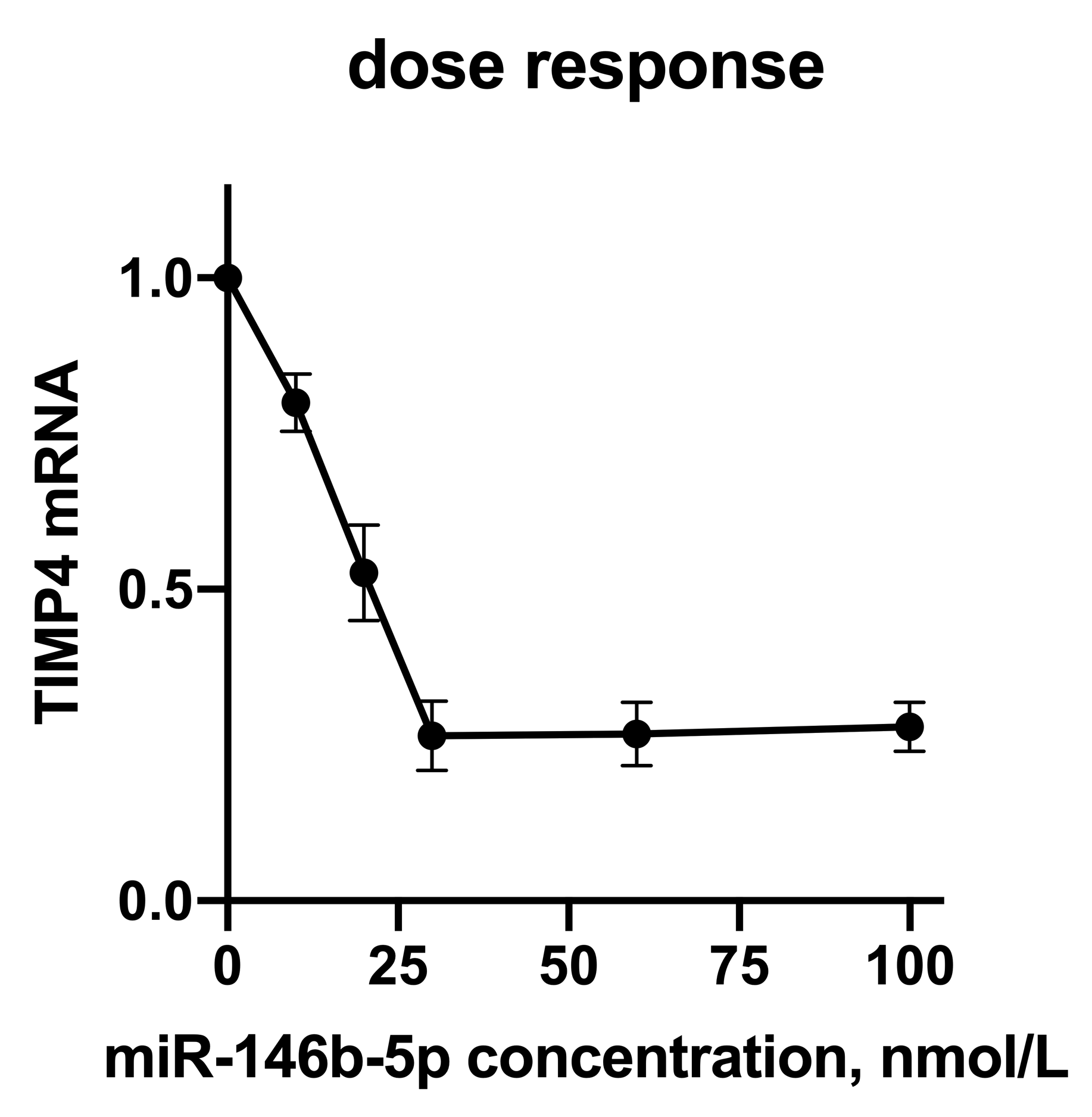

Supplemental Figure 6** Dose-response curve showing the effect of various concentrations of miR-146b-5p on the expression of exogenous TIMP4 mRNA. The minimal dose with the maximum effect is 30 nmol/L.

**
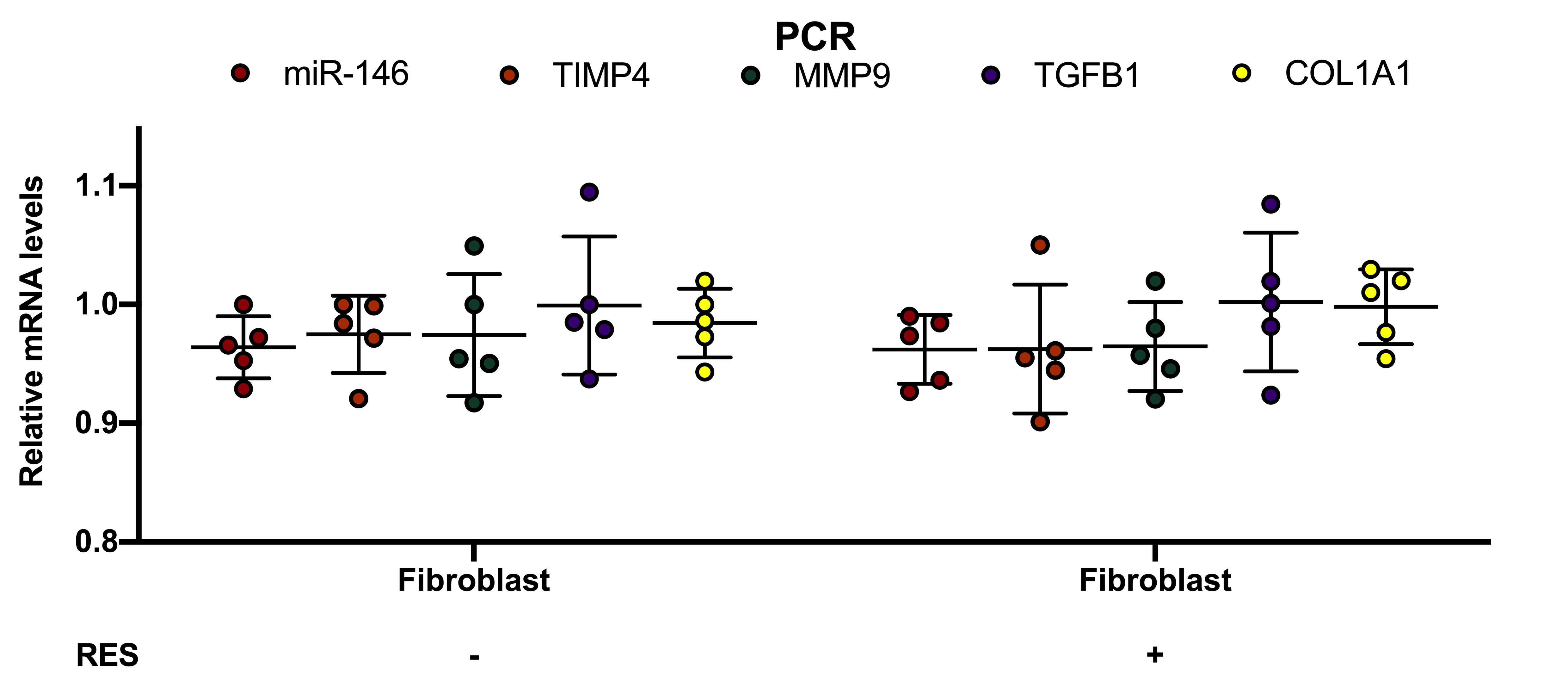
**

**Supplemental Figure 7.** RES did not change mRNA expression in the fibroblast-only culture. RES, rapid electrical field stimulation.


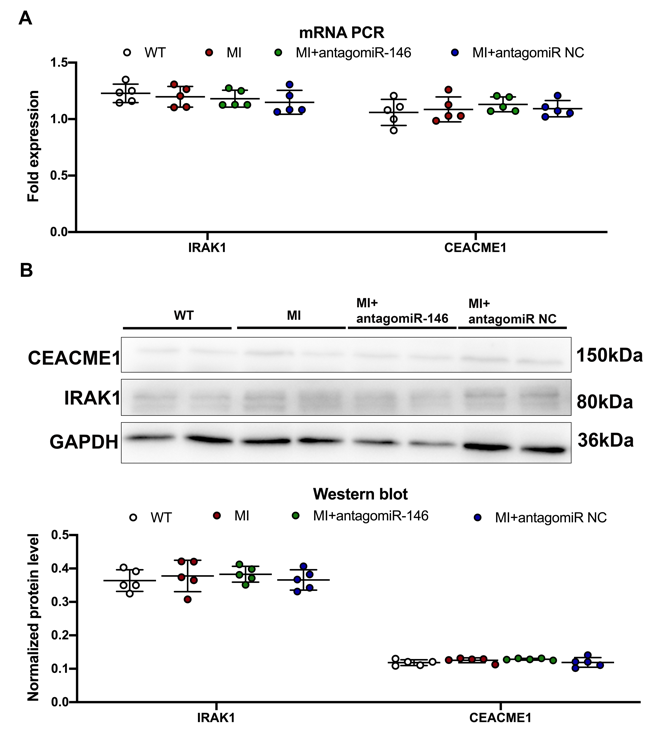


**Supplemental Figure 8.** Representative of mRNA expression **(A)** and protein expression **(B)** of IRAK1 and CEACME1 in mice. MI, myocardial infarction; WT, wild-type
